# The value of elevated cerebrospinal fluid lactate concentrations in post-neurosurgical bacterial meningitis

**DOI:** 10.1186/s12883-023-03428-8

**Published:** 2023-10-20

**Authors:** Qin Wang, YongFang Wang, Yan Yang, YanXi Kong, Yuan Peng

**Affiliations:** Intensive Care Unit, The First People ’ s Hospital of Kunshan Affiliated to JiangSu University, Kunshan, 215300 Jiangsu P.R. China

**Keywords:** Cerebrospinal fluid, Cerebrospinal fluid lactate, Cerebrospinal fluid parameters, Post-neurosurgical bacterial Meningitis, Diagnostic value

## Abstract

Bacterial meningitis is a serious complication after neurosurgery and has a high mortality rate. Early diagnosis and timely treatment are crucial or will have disastrous consequences. The classic triad of bacterial meningitis lacks sensitivity and specificity. Therefore, the diagnosis of post-neurosurgical bacterial meningitis relies on cerebrospinal fluid. But traditional cerebrospinal fluid parameters are not completely reliable in diagnosing post-neurosurgical bacterial meningitis. Previous studies have found that CSF lactate concentration is related to bacterial meningitis. But, after the craniocerebral operation, the cerebrospinal fluid of most patients is bloody. Whether red blood cells interfere with diagnosing PNBM based on lactate concentration is limited. In the current study, we further analysis on whether red blood cells interfere with diagnosing PNBM based on lactate concentration. This study aimed to investigate the value of cerebrospinal fluid lactate concentrations in post-neurosurgical bacterial meningitis. A prospective observational study was performed on 62 patients at Kunshan First People’s Hospital’s intensive care unit affiliated with Jiangsu University. We found that erythrocytes do not affect cerebrospinal fluid lactate, and elevated lactate concentrations can be used as a marker for postoperative bacterial meningitis.

**Supplementary Information:**

The online version contains supplementary material available at 10.1186/s12883-023-03428-8.

## Introduction

Approximately 120 cases of bacterial meningitis occur worldwide every year [1]. Bacterial meningitis is a serious complication after neurosurgery and has a high mortality rate [2, 3]. The incidence of intracranial infection after neurosurgery in China is 2.6%, and the mortality rate is as high as 21% [4]. However, the incidence observed in clinical practice may be higher than this reported rate. Therefore, early diagnosis and timely treatment are crucial, or disastrous consequences will ensue [5].

The classic triad of bacterial meningitis includes fever, neck stiffness, and mental state changes. This triad lacks sensitivity and specificity, and many patients do not have these three characteristics [6, 7]. Therefore, diagnosis relies on cerebrospinal fluid(CSF). However, aseptic inflammatory reactions induced by blood, bone chips, sloughing tissues, and surgical implants during surgery can simulate changes in CSF of bacterial meningitis, and the administration of prophylactic antibiotics increases the difficulty of diagnosing bacterial meningitis through routine cerebrospinal fluid analysis and culture [8–11]. Moreover, some patients with bacterial meningitis have normal or slightly elevated protein levels [12–14]. Iatrogenic hyperglycemia can mask true CSF glucose levels [15], and applying prophylactic antibiotics will also affect CSF glucose levels [16]. Gram staining of the sample can identify bacteria, but negative results cannot exclude bacterial meningitis, especially if antibiotics have been used before sampling. Therefore, traditional cerebrospinal fluid parameters are not completely reliable in diagnosing PNBM.

An ideal marker should be highly sensitive. When CSF infection exists, leukocytes increase anaerobic glucose metabolism, producing lactic acid and reducing pH. Bacterial metabolism, neutrophil glycolysis and anaerobic metabolism in brain tissues during bacterial meningitis lead to lactate accumulation in CSF [17–20]. Lactate cannot cross the blood-brain barrier to reduce the clearance rate, consequently representing a useful brain metabolic indicator [17–20]. Previous studies have found that CSF lactate concentration is related to bacterial meningitis. Two meta-analyses of 25 studies (1692 patients) and 31 studies (1885 patients) showed that the diagnostic accuracy of CSF lactate in distinguishing bacterial meningitis from aseptic meningitis is better than cerebrospinal fluid leukocyte count as well as glucose and protein concentration [21, 22]. The recently published clinical practice guidelines of the American Institute of Infectious Diseases (IDSA) for medical-related intraventricular inflammation and meningitis noted that elevated CSF lactate levels are helpful in diagnosing medical-related bacterial intraventricular inflammation and meningitis [23]. Pedro’s studies have shown that lactate is a useful tool for diagnosing PNBM [24].However, there are many patients with bloody cerebrospinal fluid after surgery, and the existing research on lactate analysis of hemorrhagic cerebrospinal fluid and non-hemorrhagic cerebrospinal fluid is limited. YuFang et al. studied different lactate levels in hemorrhagic cerebrospinal fluid and nonhemorrhagic cerebrospinal fluid [25], but there was no further analysis on whether red blood cells interfere with the diagnosis of PNBM based on lactate concentration. Therefore, this study will analyze the value of cerebrospinal fluid lactate for PNBM from the qualitative aspect of red blood cells.

### Patients and methods

#### Study design

Prospective observational diagnostic study.

### Primary objective

To evaluate the diagnosis value of CSF lactate as a marker in postsurgical patients.

#### Second objective

①To evaluate whether red blood cells affect cerebrospinal fluid lactate.

②To evaluate the correlation of CSF lactate with other CSF markers in PNBM.

③To describe the optimal CSF lactate cut-off value.

### Patients and setting

62 patients undergoing craniocerebral operation from February 2018 to February 2020 in the intensive care unit of Kunshan First People’s Hospital affiliated with Jiangsu University were selected. The study complied with the Helsinki Declaration and local laws. These postoperative patients all were suspected of having bacterial meningitis. According to the 2008 diagnostic criteria of PNBM of the United States Centers for Disease Control and Prevention/National Care Safety Network (CDC/NHSN) [26] These diagnostic criteria include clinical manifestations such as altered consciousness, neck stiffness, and fever, which are commonly associated with meningitis. Additionally, the patients’ postoperative condition and the presence of risk factors for infection, such as surgical wound site infections or immunocompromised status, contributed to the suspicion of PNBM. To confirm or rule out the diagnosis, further laboratory tests and cerebrospinal fluid analysis were conducted to identify potential pathogens and assess inflammatory markers. This comprehensive evaluation was crucial in determining the likelihood of bacterial meningitis and guiding appropriate treatment decisions.Patients were divided into the PNBM and non-PNBM groups. According to the qualitative results of red blood cells, the patients were divided into two groups: hemorrhagic cerebrospinal fluid and non-hemorrhagic cerebrospinal fluid. When the patient has a fever, headache, neck stiffness, and other unexplained changes in consciousness level, a lumbar puncture is performed to obtain cerebrospinal fluid samples, and the cerebrospinal fluid lactate test is immediately performed at the bedside. Cerebrospinal fluid samples are sent to the central laboratory for routine biochemical examination of cerebrospinal fluid, including leukocyte counts, red blood cell counts, protein and glucose concentrations, Gram staining, and bacterial culture. Patient demographic data, including age, gender, APACHEII score, GCS score, lumbar puncture time, and etiology, were collected.

Patients are classified according to predefined definitions. According to qualitative erythrocyte results in CSF, the patients were divided into hemorrhagic CSF and non-hemorrhagic CSF groups. Patients were divided into the PNBM and non-PNBM groups according to the PNBM standard formulated by CDC/NHS in 2008.

In this study, all patients underwent emergency surgery and received routine prophylactic antibiotics before and after craniotomy, using first-generation cephalosporin.

### Statistical analysis

The measurement variables are expressed by the median (interquartile interval). Comparisons between the two groups are performed using Student’s t-test for variables with a normal distribution and Wilcoxon two-sample test for variables with a non-normal distribution. Classification variables are expressed by frequency (%) and comparisons between groups are made by chi-square test or Fisher’s exact method. Mann-Whitney tests were used to estimate the area of the ROC curve where infection occurred, and the cutoff value was used when the index reached the maximum. Both used bilateral tests and statistical analysis software SAS(Version 9.3; SAS Institute Inc.,Cary, NC, USA) were used. The difference was considered statistically significant when < 0.05.

## Results

During the 2-year study period, there were 62 suspected cases of PNBM(Table 1). There were 33 males (53.2%) and 29 females (46.8%). In total, 23 cases of brain injury and 39 cases of spontaneous cerebral hemorrhage were diagnosed on admission. This study included 38 cases with hemorrhagic cerebrospinal fluid and 24 cases with non-hemorrhagic cerebrospinal fluid. The diagnosis was PNBM in 43 cases and non-PNBM in 19 cases. There were no significant differences in age, sex, admission diagnosis, and APACHEII score among the groups (p > 0.05).

**Table 1:** Demographic data

Group	Hemorrhagic CSF (n = 38)			Non-hemorrhagic CSF (n = 24)	
	PNBM group (n = 29)	Non-PNBM group (n = 9)		PNBM group (n = 14)	Non-PNBM group (n = 10)
Age, median and range ( year)	53(43–65)	46 (40–51)		46(37–53)	42 (37–46)
Gender (%)					
Male	18(62.1)	6 (66.7)		5 (35.71)	4 (40.0)
Female	11 (37.9)	3 (33.3)		9 (64.29)	6 (60.0)
Indication for surgery (%)					
Brain injury	17 (58.6)	4 (44.4)		6 (42.9)	3 (30.0)
Intracranial bleeding	12 (41.4)	5 (55.6)		8 (57.1)	7 (70.0)
APACHE II score	20(16–21)	18 (16–18)		17(15–18)	15 (14–15)
Lumbar Puncture (days after surgery)	7 (6–10)	7 (7–7)		8 (6–10)	7 (6–9)

Abbreviation: PNBM:post-neurosurgical bacterial meningitis; APACHE II: Acute Physiology and Chronic Health Evaluation

To analysis on whether red blood cells interfere with diagnosing PNBM based on lactate concentration. We compared cerebrospinal fluid parameters between hemorrhagic and non-hemorrhagic cerebrospinal fluid groups. We found that lactate concentration (5.3 mmol/L vs. 5.0 mmol/L, P > 0.05) and protein level (171.5 mg/dl vs. 151 mg/dl, p > 0.05) are increased in the hemorrhagic cerebrospinal fluid group compared with the non-hemorrhagic cerebrospinal fluid group; In contrast, the glucose concentration (2.1 mmol/L vs. 2.5 mmol/L, P > 0.05) is lower in the hemorrhagic cerebrospinal fluid group compared with the non-hemorrhagic cerebrospinal fluid group. Still, the difference is not statistically significant. While the white blood cell count in the two groups is different(349 *109/L vs. 115 *109/L, p < 0.05), the difference is statistically significant. The difference in blood glucose concentration (8.632 mmol/L in Hemorrhagic CSF group vs. 8.554 mmol/L in Non-hemorrhagic CSF group) and blood lactate concentration (2.387 mmol/L in Hemorrhagic CSF group vs. 2.238 mmol/L in Non-hemorrhagic CSF group) between the two groups was not statistically significant (P = 0.879 and P = 0.376, respectively). (Table 2). The results showed that CSF erythrocytes did not affect CSF lactate, glucose, and protein concentrations. However, the count of CSF leukocytes in the hemorrhagic CSF group is increased compared with the non-hemorrhagic CSF group, and the difference is statistically significant (P < 0.05). These results indicate that cerebrospinal fluid lactate is not affected by erythrocytes.

**Table 2:** Comparison of cerebrospinal fluid parameters between hemorrhagic and nonhemorrhagic cerebrospinal fluid groups

Group	Hemorrhagic CSF (n = 38)	Non-hemorrhagic CSF (n = 24)	P
Lactate level ( mmol/L)	5.35 (4.30–6.80)	5.00 (3.25–6.25)	0.174
WBC ( ×10^6^/L)	349.0(146–2648)	115.0 (32.0-1038.5)	0.021
Glucose level ( mmol/L)	2.15 (1.70–2.30)	2.50 (1.75–2.70)	0.069
Protein level ( mg/dl)	171.5(70.0-294.0)	151(54–270)	0.435
Blood glucose concentration (mmol/L)	8.632 ± 2.150	8.554 ± 1.570	0.879
Blood lactate concentration (mmol/L)	2.387 ± 0.674	2.238 ± 0.588	0.376

Abbreviation: WBC: white blood cell; CSF:cerebrospinal fluid

Then, we compared the cerebrospinal fluid parameters between the PNBM and non-PNBM groups. Lactate levels (6.1 mmol/L vs. 3.5 mmol/L, p < 0.05)and protein concentrations(222 mg/dl vs. 54 mg/dl, p < 0.05) in the PNBM group were increased compared with the non-PNBM group, whereas the glucose concentration (2.1 mmol/L vs. 2.8 mmol/L, p < 0.05) in the PNBM group was lower than that in a non-PNBM group. The blood glucose concentration (8.351 ± 2.091 mmol/L vs. 9.168 ± 1.402 mmol/L, P = 0.126) did not show a statistically significant difference between the PNBM group and the Non-PNBM group, while the blood lactate concentration was significantly higher in the PNBM group (2.458 ± 0.590 mmol/L) compared to the Non-PNBM group (2.037 ± 0.671 mmol/L) (P = 0.016)(Table 3). The difference was statistically significant (p < 0.05). The results showed that the CSF lactate level and protein and glucose concentrations mainly reflected the infection.

**Table 3:** Comparison of cerebrospinal fluid parameters between the PNBM group and non-PNBM group

Group	PNBM group (n = 43)	Non-PNBM group (n = 19)	P
Lactate level ( mmol/L)	6.1 (5.3–7.1)	3.5(3.1–4.1)	< 0.001
WBC ( ×10^6^/L)	631.0(206.0-2567.0)	32.00(23.0–43.0)	< 0.001
Glucose level ( mmol/L)	2.1(1.6–2.3)	2.8 (2.5–3.1)	< 0.001
Protein level ( mg/dl)	222.0(160.0-300.0)	54.0(26.0–59.0)	< 0.001
Blood glucose concentration (mmol/L)	8.351 ± 2.091	9.168 ± 1.402	0.126
Blood lactate concentration (mmol/L)	2.458 ± 0.590	2.037 ± 0.671	0.016

For both the Hemorrhagic CSF group and the Non-hemorrhagic CSF group, there were significant differences in lactate levels, white blood cell counts, glucose levels, and protein levels between the PNBM group and the Non-PNBM group (all P-values < 0.001). The difference in blood glucose concentration was not significant in the PNBM group (P = 0.087) or in the Non-PNBM group (P = 0.851). However, there was a significant difference in blood lactate concentration within the PNBM group (P = 0.004), while no significant difference was observed in the Non-PNBM group (P = 0.271) (Supplementary Table 1).

**Fig. 1:**
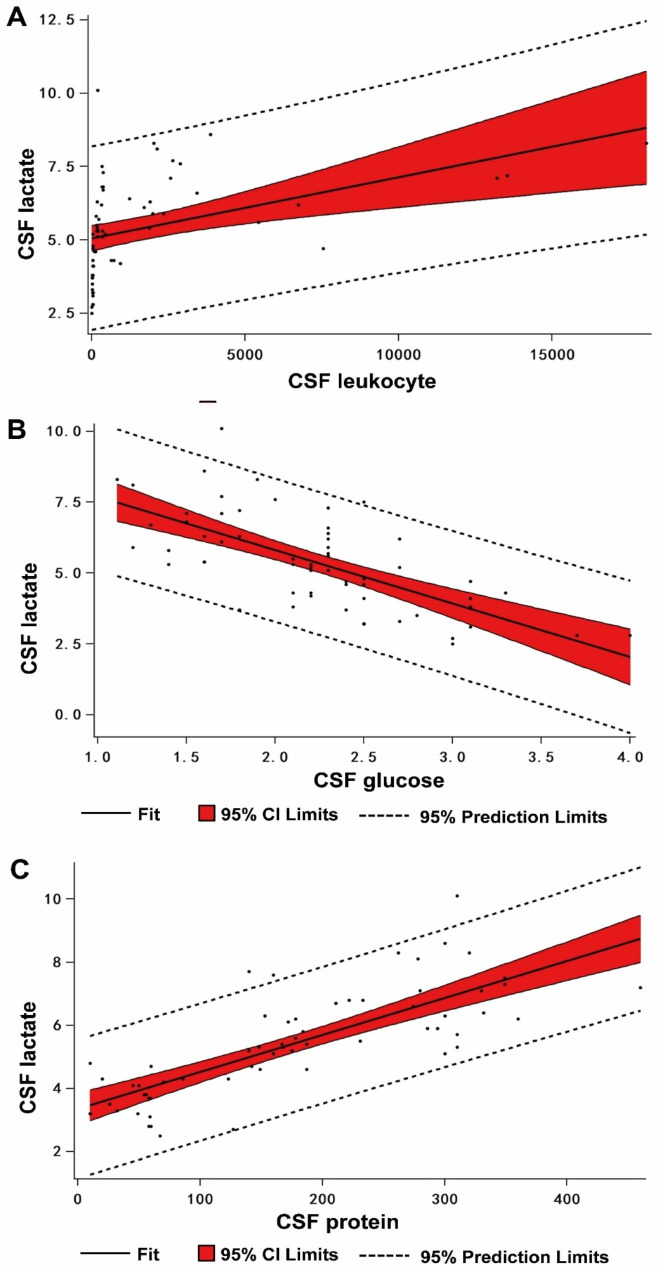
The correlation analysis of CSF lactate and other parameters. **A**:The correlation analysis between CSF lactate and CSF leukocyte. **B**: The correlation analysis betweenCSF lactate and CSF glucose. **C**:The correlation analysis between CSF lactate and CSF protein

We also evaluated the correlation between CSF lactate and other parameters(Fig. 1). According to the Person correlation coefficient, cerebrospinal fluid lactate concentration exhibits a good correlation with CSF glucose and CSF protein, and its correlation coefficients are (-0.68; P < 0.001 vs. 0.77; P < 0.001), respectively. In contrast, cerebrospinal fluid lactate concentration is moderately correlated with white blood cell count (r = 0.42; P < 0.001).

**Fig. 2:**
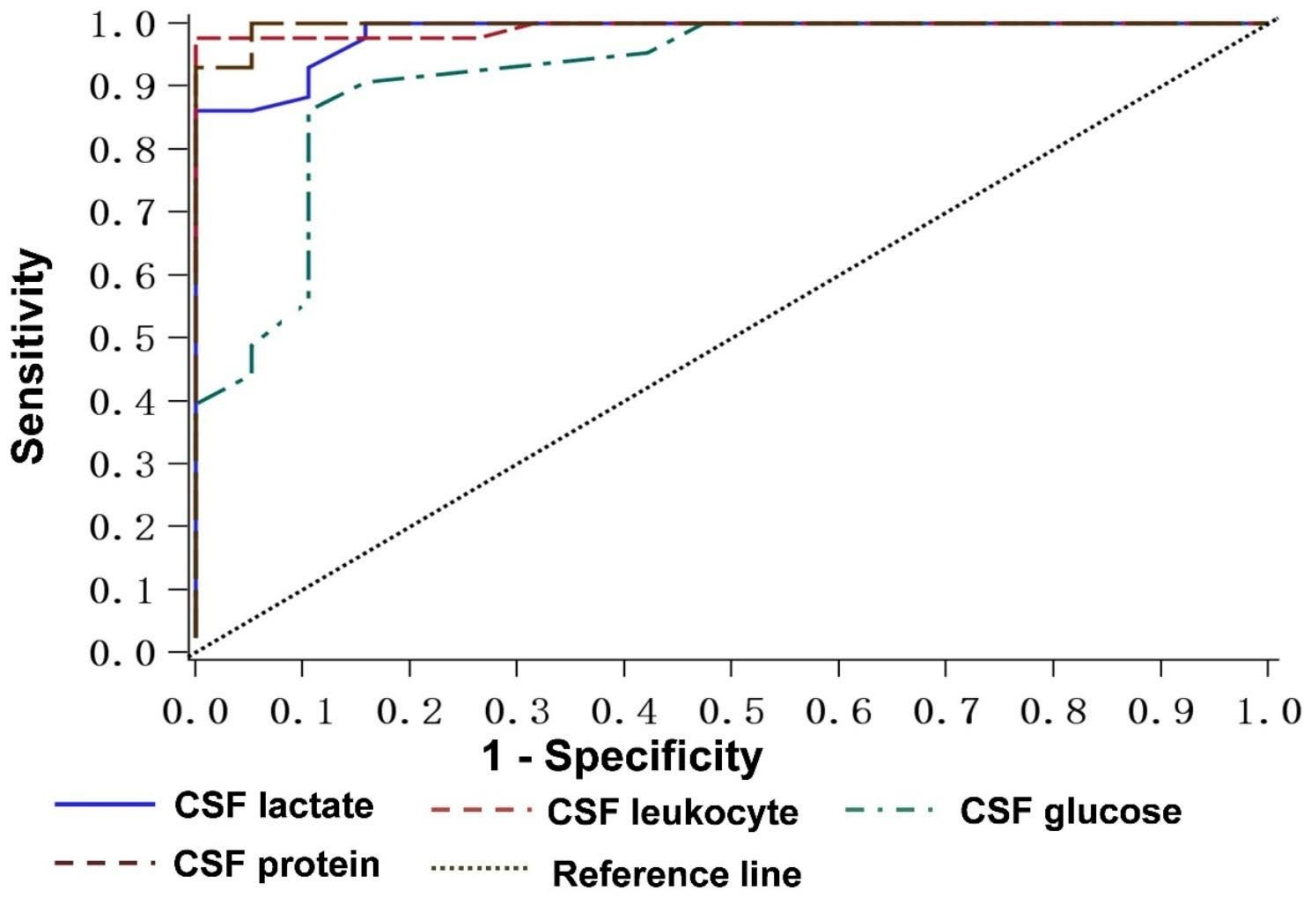
ROC curve of diagnostic effectiveness of CSF parameters in PNBM

ROC curve analysis of CSF parameters shows that CSF lactate levels exhibit good diagnostic efficiency (AUC 0.98, 95% CI 0.96-1.00%)(Fig. 2). When the CSF lactate threshold is 4.95 mmol/L, the sensitivity is 86%, and the specificity is 100%.

## Discussion

Cerebrospinal fluid acidosis caused by bacterial infection is well described. When CSF infection exists, leukocytes increase anaerobic glucose metabolism, producing lactic acid and reducing pH. Bacterial metabolism, neutrophil glycolysis, and anaerobic metabolism of brain tissues during bacterial meningitis lead to CSF lactate accumulation [17–20]. Lactate does not cross the blood-brain barrier to reduce the clearance rate, consequently representing a useful brain metabolic indicator [17–20]. The CSF lactate test is simple, convenient, and affordable. The test can be performed at the bedside, and the results are obtained within a few minutes.

After the craniocerebral operation, the cerebrospinal fluid of most patients is bloody due to primary disease, operation, or lumbar puncture injury. The presence of red blood cells in CSF will lead to an increase in lactate levels [27]. This result has been recorded in previous simulated hemorrhagic cerebrospinal fluid [28] and animal models [29]. However, Maskin’s study showed that erythrocytes in cerebrospinal fluid did not affect lactate levels [30]. To evaluate whether red blood cells influence lactate levels, this study compared the difference in CSF lactate levels between the hemorrhagic CSF group and the non-hemorrhagic CSF group. Lactate levels in the hemorrhagic CSF group were increased compared with the non-hemorrhagic CSF group, but the difference was not statistically significant. These results indicate that erythrocytes in CSF did not affect lactate levels. Comparing other cerebrospinal fluid parameters in the hemorrhagic and non-hemorrhagic CSF groups shows that the white blood cell count in the hemorrhagic CSF group is increased compared with the non-hemorrhagic CSF group, and the difference is statistically significant. These results indicate that white blood cell counts should not be used as a reference marker for diagnosing PNBM in hemorrhagic CSF. To confirm that white blood cell counts do not affect CSF lactate levels, Pearson correlation analysis was performed to compare the possible effects of white blood cell counts on lactate in specimens. We observed a moderate correlation between leukocyte count and CSF lactate (r = 0.42). Tavares et al. [31] assessed the value of different CSF parameters in diagnosing PNBM and found that CSF glucose (AUC: 0.85) and lactate (AUC: 0.85) are good differential markers of bacterial infection. We reported similar findings. In the correlation analysis between CSF lactate and glucose, CSF lactate was strongly correlated with glucose (r=-0.68). We further compared the differences in CSF lactate between the PNBM group and the non-PNBM group. We found that the lactate value in the PNBM group was increased compared with the non-PNBM group, and the difference was statistically significant. These results indicate that infection affected lactate levels. The increase in CSF lactate levels mainly reflects the infection, which is more consistent with our need to diagnose PNBM.

Previous studies have also discussed the relationship between cerebrospinal fluid lactate levels and mortality in patients with meningitis. Abassi et al [32]. found that cerebrospinal fluid lactate levels > 5 mmol/L were independently associated with an increased risk of excess mortality (adjusted hazard ratio = 3.41; 95% confidence interval, 1.55–7.51; P = 0.002). Elevated cerebrospinal fluid lactate levels were also found to be correlated with altered mental status and seizure occurrence in these individuals. In our study, the average cerebrospinal fluid lactate concentration in the PNBM group was 6.1 mmol/L, significantly higher than the Non-PNBM group. Therefore, it is crucial to establish targeted therapeutic management strategies for patients with elevated cerebrospinal fluid lactate levels. Furthermore, Almeida et al [33]. also identified increased lactate concentration as a predictor of adverse outcomes, indicating that dynamic monitoring of cerebrospinal fluid lactate levels should be enhanced as it relates to the prognosis of patients with meningitis. Overall, the studies suggest that cerebrospinal fluid lactate levels have prognostic significance in meningitis patients and may serve as an important marker for assessing disease severity and guiding appropriate management strategies.

Leib et al. [34] first retrospectively analyzed 77 patients after neurosurgery according to clinical diagnostic criteria and found that the sensitivity and specificity of diagnosis were 88% and 98%, respectively, when the cut-off value of CSF lactate was 4 mmol/L. Tavares et al. [31] analyzed 28 post-neurosurgical patients and found that when the cerebrospinal fluid lactate critical value was 49 milligrams per deciliter (mg/dL), the sensitivity and specificity of the diagnosis were 86% and 90.5%, respectively. This finding may be helpful in distinguishing between bacterial meningitis and aseptic meningitis. Grill et al. [35] evaluated the CSF lactate level using clinical standards and gold standards and found that CSF lactate exhibited diagnostic accuracy. Subsequently, Xiao et al.’ s [36] meta-analysis of five studies, including 404 neurosurgical patients, showed that CSF lactate diagnosis of PNBM has high sensitivity and specificity. In our study, CSF lactate has a good diagnostic effect on PNBM, which was treated with antibiotics and was cerebrospinal fluid culture negative. When the diagnostic threshold is 4.95 mmol/L, the sensitivity is 86%, and the specificity is 100%, consistent with Maskin et al.‘s research results. [30] and other authors [37, 38].

In our study, we observed that the average blood lactate and blood glucose concentrations are higher in patients of the PNBM group. Zheng et al [39]., in a multicenter study based on routine clinical cerebrospinal fluid examinations, developed a model to predict PNBM. They concluded that combining cerebrospinal fluid/blood glucose ratio and blood glucose concentration can improve the predictive efficacy of PNBM. Similarly, Zhang et al [40]. found that relying solely on blood routine examinations for predicting PNBM had limited accuracy and suggested incorporating variables like cerebrospinal fluid lactate to enhance predictive ability. Currently, there is limited research on using blood lactate concentration to predict PNBM, and future studies should increase sample sizes to conduct comprehensive analysis combining blood lactate and cerebrospinal fluid lactate to predict PNBM.This study also has some limitations. We chose clinical criteria to diagnose PNBM, but positive cerebrospinal fluid culture or gram stain results are the gold standards for diagnosis. Due to the overlapping symptoms and signs of PNBM with other diseases, as well as the potential influence of factors such as the patient’s immune status, age, and underlying conditions, clinical diagnosis may not always be 100% accurate. In the absence of bacterial cultures or Gram staining as laboratory confirmatory tests, the precision of diagnosing PNBM could be compromised. Nonetheless, despite its limitations, clinical suspicion remains a crucial basis for timely intervention, particularly in acute conditions like infectious meningitis, where rapid diagnosis and treatment are of paramount importance for the patient’s prognosis. A more comprehensive understanding of the diagnosis and treatment of PNBM can be achieved in the future through further research into different diagnostic methods, including laboratory tests and imaging techniques. However, patients received prophylactic antibiotic administration before and after surgery due to emergency surgery. Regarding other various reasons, the cerebrospinal fluid cultures of all patients in the study were negative, so we chose clinical criteria to diagnose PNBM. However, this standard is quite authoritative, so we can confidently use it. In addition, the relatively small sample size also needs to be improved in this study. The clinical efficiency and safety of using CSF lactate levels to exclude PNBM must be verified in a larger population.

Our research shows that CSF lactate mainly reflects infection and is not related to the number of red blood cells in cerebrospinal fluid. CSF lactate concentration is a reliable indicator for the diagnosis of PNBM. When the diagnostic threshold of CSF lactate is 4.95 mmol/L, the specificity is 100%, which is useful for excluding bacterial meningitis after neurosurgery under appropriate clinical conditions.

### Electronic supplementary material

Below is the link to the electronic supplementary material.

Supplementary Material 1

## Data Availability

The datasets generated and analysed during the current study are available from the corresponding author on reasonable request.

## Abbreviations

CSF: cerebrospinal fluid
PNBM: post-neurosurgical bacterial meningitis
IDSA: Institute of Infectious Diseases
