# Supplementary material for: The value of elevated cerebrospinal fluid lactate concentrations in post-neurosurgical bacterial meningitis
Source: BMC Neurol. 2023 Oct 20;23:377. doi: 10.1186/s12883-023-03428-8 (PMC10588014; doi:10.1186/s12883-023-03428-8)
Supplement: Supplementary file 1 — Supplementary Material 1 [file 12883_2023_3428_MOESM1_ESM.docx]

Supplementary Table 1. Subgroup Analysis Based on the Presence of Hemorrhagic Cerebrospinal Fluid

|  | Hemorrhagic CSF | |  | Non-hemorrhagic CSF | |  |
| --- | --- | --- | --- | --- | --- | --- |
| Group | PNBM group | Non-PNBM group | P | PNBM group | Non-PNBM group | P |
| Lactate level ( mmol/L) | 6.2 (5.1-7.4) | 3.6(3.0-4.3) | <0.001 | 6.0(5.4-7.0) | 3.4(2.9-4.0) | <0.001 |
| WBC ( ×10^6^/L) | 712.0(308.0-2446.0) | 38.0(27.0-49.0) | <0.001 | 411.0(180.0-1310.5) | 35.0(27.0-45.0) | <0.001 |
| Glucose level ( mmol/L) | 2.0(1.7-2.6) | 3.0 (2.2-3.5) | <0.001 | 2.3(2.0-3.6) | 2.6(1.8-3.2) | <0.001 |
| Protein level ( mg/dl) | 241.0(170.0-307.0) | 51.0(28.0-61.0) | <0.001 | 170.0(32.0-280.0) | 30.0(24.0-56.0) | <0.001 |
| Blood glucose concentration (mmol/L) | 8.400±2.362 | 9.378±1.012 | 0.087 | 8.250±1.445 | 8.980±1.713 | 0.851 |
| Blood lactate concentration (mmol/L) | 2.555±0.617 | 1.844±0.577 | 0.004 | 2.257±0.491 | 2.210±0.731 | 0.271 |
